# Predicting Recurrence in Locally Advanced Rectal Cancer Using Multitask Deep Learning and Multimodal MRI

**DOI:** 10.1148/rycan.240359

**Published:** 2025-05-30

**Authors:** Zonglin Liu, Runqi Meng, Qiong Ma, Zhen Guan, Rong Li, Caixia Fu, Yanfen Cui, Yiqun Sun, Tong Tong, Dinggang Shen

**Affiliations:** ^1^Department of Radiology, Fudan University Shanghai Cancer Center, 270 Dongan Rd, 270, Xuhui District, Shanghai, 200032, China; ^2^Department of Oncology, Shanghai Medical College, Fudan University, Shanghai, China; ^3^School of Biomedical Engineering & State Key Laboratory of Advanced Medical Materials and Devices, ShanghaiTech University, Shanghai, China; ^4^Department of Radiology, Key Laboratory of Carcinogenesis and Translational Research (Ministry of Education/Beijing), Peking University Cancer Hospital and Institute, Beijing, China; ^5^MR Application Development, Siemens Shenzhen Magnetic Resonance, Shenzhen, China; ^6^Guangdong Provincial Key Laboratory of Artificial Intelligence in Medical Image Analysis and Application, Guangzhou, China; ^7^Department of Radiology, Shanxi Province Cancer Hospital/Shanxi Hospital Affiliated to Cancer Hospital, Chinese Academy of Medical Sciences/Cancer Hospital Affiliated to Shanxi Medical University, Shanxi, China; ^8^Shanghai United Imaging Intelligence, Shanghai, China; ^9^Shanghai Clinical Research and Trial Center, Shanghai, China

**Keywords:** MR-Imaging, Abdomen/GI, Rectum, Oncology

## Abstract

**Purpose:**

To develop and validate a deep multitask network, MultiRecNet, for fully automatic prediction of disease-free survival (DFS) in patients with neoadjuvant chemoradiotherapy (nCRT)–treated locally advanced rectal cancer (LARC).

**Materials and Methods:**

This retrospective study collected clinical information and baseline multimodal MRI (T2, apparent diffusion coefficient [ADC], *D*_app_, and *K*_app_) data from patients with LARC after nCRT at three centers between October 2011 and May 2019. Patients from centers 1 and 2 were divided into training, validation, and internal testing sets, while patients from center 3 served as the external testing set. MultiRecNet is capable of simultaneously performing segmentation, classification, and survival prediction tasks within a single framework. Multiple combinations of data from different clinical stages (pretreatment and postoperative) were input into MultiRecNet to generate different models and identify the model with optimal performance. Evaluation metrics included the Dice similarity coefficient (DSC), the area under the receiver operating characteristic curve (AUC), and the Harrell concordance index (C-index) for the segmentation, classification, and survival prediction tasks, respectively.

**Results:**

The study included 445 patients: 261 in the training set (median age, 60 years [IQR, 53–67 years]; 172 male), 37 in the validation set (median age, 61 years [IQR, 55–68 years]; 30 male), 75 in the internal testing set (median age, 60 years [IQR, 51–67 years]; 45 male), and 72 in the external testing set (median age, 55 years [IQR, 49–61 years]; 38 male). In the internal testing set, the best model based on MultiRecNet (the All model, with T2-weighted imaging, ADC, *D*_app_, *K*_app_, pretreatment clinical indicators, and postoperative pathologic indicators) achieved a DSC of 0.72 for tumor segmentation, an AUC of 0.97 (95% CI: 0.92, >.99) for recurrence or metastasis classification at 3 years, and a C-index of 0.92 for DFS prediction. In the external testing set, the model continued to perform well for survival prediction (C-index = 0.81, *P* < .001).

**Conclusion:**

The MultiRecNet-based model enabled prognostic prediction in a fully automated end-to-end manner in patients with LARC following nCRT.

**Keywords:** MR-Imaging, Abdomen/GI, Rectum, Oncology

*Supplemental material is available for this article.*

Published under a CC BY 4.0 license.

## Graphical abstract

**Figure:**
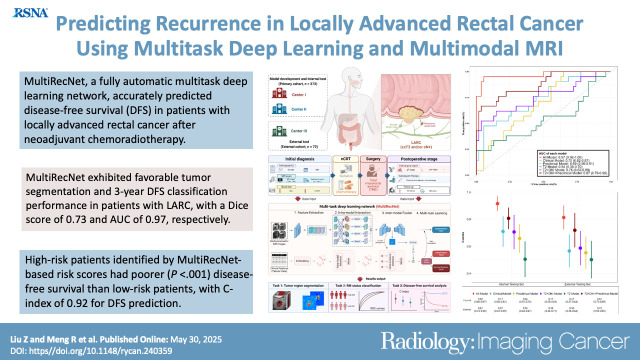

**Summary**

MultiRecNet, a fully automatic multitask deep learning network, accurately predicted disease-free survival in patients with locally advanced rectal cancer after neoadjuvant chemoradiotherapy, using multimodal MRI.

**Key Points**

■ MultiRecNet, a fully automatic multitask deep learning network, exhibited favorable tumor segmentation and 3-year recurrence or metastasis classification performance in patients with locally advanced rectal cancer in both the initial diagnostic and postoperative stages (with Dice similarity coefficient of 0.73 and 0.72, respectively, for tumor segmentation; area under the receiver operating characteristic curve of 0.87 and 0.97, respectively, for classification performance) in the internal testing set.
■ High-risk patients identified by MultiRecNet-based risk scores had poorer (*P* < .001) disease-free survival than low-risk patients in the initial diagnostic and postoperative stage both in the internal testing set (Harrell concordance index [C-index] of 0.81 and 0.92, respectively) and external testing set (C-index of 0.72 and 0.81, respectively).

## Introduction

According to the National Comprehensive Cancer Network guidelines, the current standard treatment protocol for patients with locally advanced rectal cancer (LARC) is total mesorectal excision combined with preoperative neoadjuvant chemoradiotherapy (nCRT), which has been proven effective for local control (1). However, variations in recurrence or metastasis (RM) and long-term disease-free survival (DFS) have remained major issues among different populations. More than 30% of patients with LARC experienced RM in the short-term postoperative period with standard treatment (2,3), while certain individuals achieved a favorable prognosis even with moderate-intensity treatment (4,5). Therefore, implementing an appropriate treatment regimen is crucial for improving the long-term DFS of patients with LARC. Early prediction of DFS can provide valuable information for guiding treatment selection, but few clinically validated tools have been developed.

Several MRI methods for evaluating DFS-related factors have been reported in patients with rectal cancer (6,7). In particular, the value of functional imaging features, such as diffusion-weighted imaging, has been emphasized (8). Diffusion kurtosis imaging (DKI), a polyexponential non-Gaussian diffusion model, yields several quantitative parameters (eg, *D*_app_ and *K*_app_). These parameters describe the extent to which the diffusion model deviates from the Gaussian distribution, providing a better measure of the complexity of the organic structure than does conventional diffusion-weighted imaging (9). Previous studies have shown that DKI parameters are significantly associated with various pathologic prognostic factors (eg, histologic grade, nodal involvement, and lymphovascular invasion) and treatment outcomes (10–14). Furthermore, Cui et al (15) reported the feasibility of predicting DFS in patients with LARC with a DKI-based radiomic model, but this approach requires labor-intensive manual delineation of the tumor mask and feature definition, limiting its clinical application.

Deep learning (DL) techniques can effectively circumvent these problems, and their potential for prognostic prediction in various tumor types has been reported (16–19). However, no-mask DL models for prognostic prediction, typically based on convolutional neural networks, may introduce interference from irrelevant background information, potentially causing the model to miss tumor-specific information. Recently, the use of multitask DL networks for segmentation and classification tasks has been explored (20–22). This approach allows the model to focus on the tumor region without losing extratumoral information due to implicit guidance patterns. Because of the multitask nature of this approach, the entire process can be automated, showing promise for clinical applications.

Therefore, we developed and validated a multitask DL network based on clinical information and multimodal MR images (T2-weighted and DKI) for DFS prediction in patients with LARC who underwent nCRT. This network simultaneously performed segmentation, risk classification, and survival prediction tasks in an end-to-end manner.

## Materials and Methods

### Ethics Statement

This retrospective, multicenter study was approved by the institutional review boards of the participating institutions, with a waiver of informed consent granted due to its retrospective nature. The study was performed in accordance with the Declaration of Helsinki, designed following the guidelines of Transparent Reporting of a multivariate prediction models for Individual Prognosis or Diagnosis (ie, TRIPOD), and classified as a type III study (23).

### Data Collection

**Figure 1:**
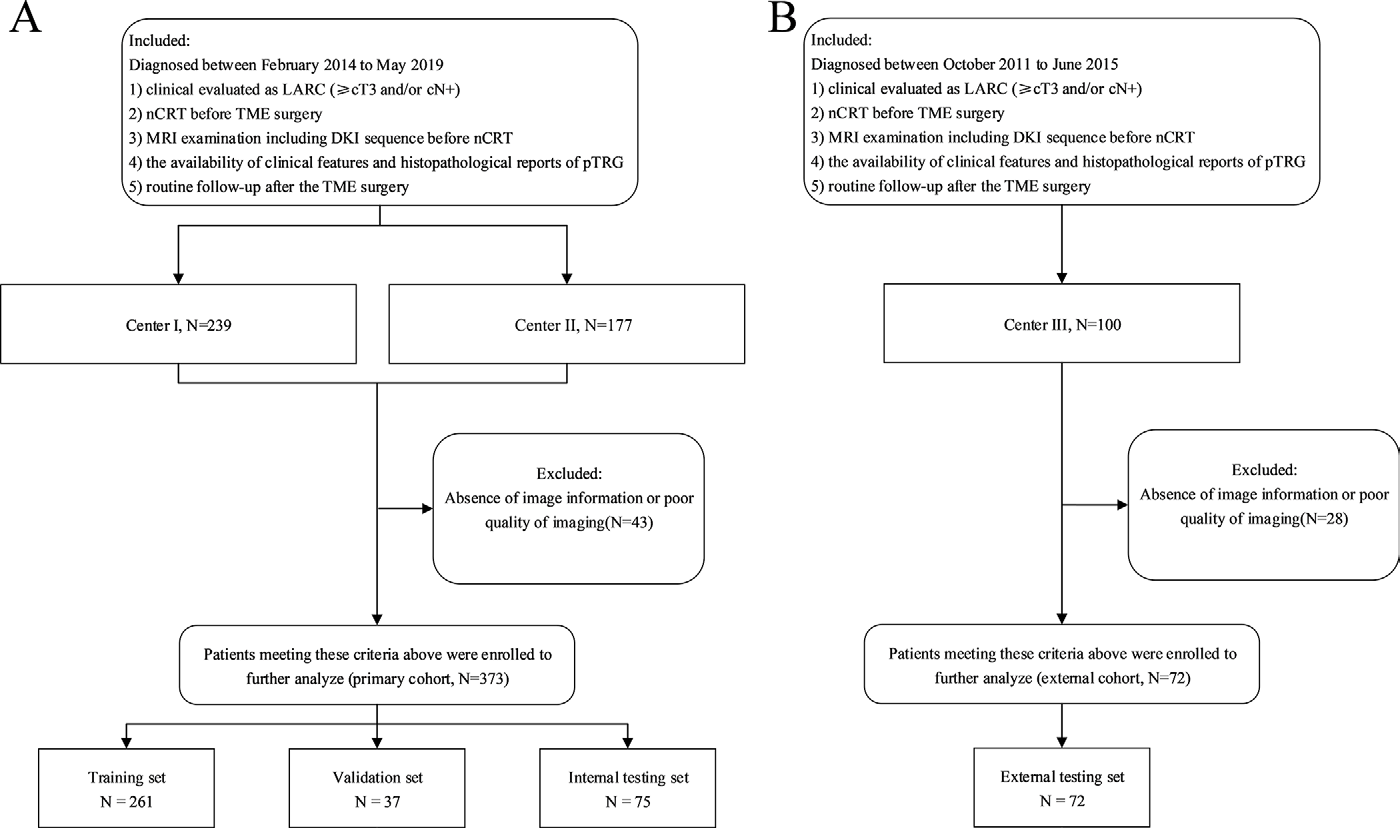
Flowcharts show patient inclusion for **(A)** the primary cohort composed of the training, validation, and internal testing sets and **(B)** the external cohort composed of the external testing set. DKI = diffusion kurtosis imaging, LARC = locally advanced rectal cancer, nCRT = neoadjuvant chemoradiotherapy, pTRG = pathologic tumor regression grade, TME = total mesorectal excision.

The study data were obtained from three institutions in China. Patients with rectal cancer from Fudan University Shanghai Cancer Center (center 1) and Shanxi Province Cancer Hospital (center 2) between February 2014 and May 2019 were included as the primary cohort. The inclusion criteria for patients were as follows: *(a)* histologically confirmed rectal adenocarcinoma, *(b)* underwent nCRT within 2 weeks before total mesorectal excision surgery, *(c)* had pretreatment MR images, including DKI sequences, available and clinically evaluated as LARC (≥cT3 and/or cN+), *(d)* had clinical features available, and *(e)* had routine follow-up after total mesorectal excision surgery. Patients without image information or with poor quality images were excluded. Then, patients from the primary cohort were randomly allocated to training, validation, and internal testing sets at a ratio of 7:1:2, which were used for model construction, hyperparameter optimization, and performance evaluation (Fig 1A).

Patients from Peking University Cancer Hospital and Institute (center 3) between October 2011 and June 2015 were included as the external cohort to evaluate the performance of our models (Fig 1B).

In addition, data regarding pretreatment clinical status (age, sex, carcinoembryonic antigen level, cancer antigen 19-9 level, location of lesion, clinical stage, clinical T and N stages, MR-detected mesorectal fascia, and MR-detected extramural venous invasion) and postoperative pathologic status (pathologic T and N stages, lymphovascular invasion, perineural invasion, and pathologic tumor regression grade) were obtained from the electronic medical records. The pTRG classification criteria were in accordance with the American Joint Committee on Cancer four-stage grading system (22). The postoperative treatment status was recorded, including information such as whether the patient underwent postoperative adjuvant chemotherapy.

All instances of RM were confirmed by histopathologic examination or follow-up imaging studies (including CT, PET/CT, or MRI) that demonstrated the development of new local or distant lesions. Patients without RM had a minimum follow-up of 36 months (3 years).

### Imaging Protocols

No bowel preparation was administered before the MRI examination. All MR images were obtained with 3-T MRI scanners (Table S1). DK images were processed to obtain *D*_app_, *K*_app_, and apparent diffusion coefficient (ADC) images on a prototype postprocessing workstation (Siemens Healthineers, as detailed in Appendix S1). These images and oblique axial thin-section T2-weighted images were subsequently transferred to a mobile workstation for manual delineation with ITK-SNAP software (version 4.0.0; *www.itk-snap.org*). Two radiologists (Z.L. and Y.S., with 3 and 10 years of clinical experience in diagnosing rectal cancer, respectively) independently delineated the volumes of interest along tumor contours on the axial T2-weighted images with reference to the ADC images, following the annotation guidelines from the Expert Consensus on Colorectal Cancer CT and MRI Annotation (2020) (24). Except for the tumor location, the radiologists were blinded to all the clinical information. All volumes of interest were validated by an additional senior expert in rectal cancer imaging with more than 16 years of clinical experience (T.T.). To achieve spatial alignment across all MRI sequences, a rigid transformation was implemented to register the *D*_app_, *K*_app_, and ADC images to the T2-weighted images (as detailed in Appendix S2).

### DL Network Development and Model Selection

**Figure 2:**
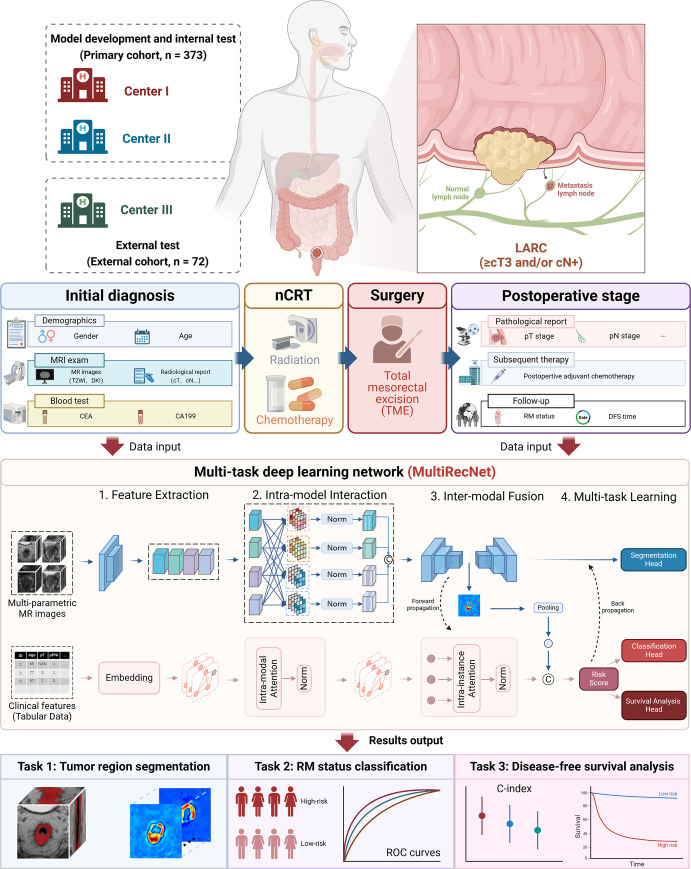
Overview of the clinical workflow, study design, and network details. A total of 373 patients with LARC from centers 1and 2 were included in the primary cohort for model development and internal testing, while 72 patients from center 3 were included for external testing. The initial diagnostic data before nCRT (including demographic data, MRI data with the volume of interest, and blood test data) and pathologic data after TME surgery were input into the multitask deep learning network, MultiRecNet. Then, three tasks were simultaneously implemented, including tumor segmentation, recurrence status classification, and survival prediction, with the final outputs. C-index = concordance index, DFS = disease-free survival, DKI = diffusion kurtosis imaging, LARC = locally advanced rectal cancer, nCRT = neoadjuvant chemoradiotherapy, RM = recurrence or metastasis, ROC = receiver operating characteristic, TME = total mesorectal excision, T2WI = T2-weighted imaging.

MultiRecNet, a multimodal imaging–based multitask DL network, was developed to predict tumor RM in patients with LARC (Fig 2). This network simultaneously predicts tumor volume, patient RM status at 3 years, and survival risk score by using multimodal MR images and clinical data. MultiRecNet incorporates elements from both three-dimensional U-Net (25) and a graph neural network (26) to extract features from image and clinical data. Several modules were introduced to enhance effectiveness and robustness, including a multimodality adaptive learning module, a masking strategy, an interinstance interaction module, and a synergized dual-stream structure (as detailed in Appendices S3 and S4). A comparative analysis was conducted to evaluate performance against various single-task and multitask approaches from previous studies. Specifically, for single-task approaches, we utilized U-Net and V-Net for tumor segmentation, while VGG16, InceptionV3, DenseNet, and ResNet were used for patient RM status prediction. For the multitask analysis, we applied the Transformer-based Multimodal network for Segmentation and Survival prediction (TMSS) (20) and Deep Multi-Task Survival model (DeepMTS) (21), which were specifically tailored for survival analysis and tumor segmentation tasks. Throughout the optimization process, we employed a combination of the focal Dice loss, cross-entropy loss, and negative log-likelihood loss, weighted at a 1:2:1 ratio (as detailed in Appendix S5). The modules and packages used in the model construction process are shown in Table S2.

To approximate real clinical use scenarios, we experimented with various types of available data at different clinical stages as inputs into MultiRecNet, including baseline images, pretreatment clinical data, postoperative pathologic data, and different data combinations. For model selection, we chose the model with the highest area under the receiver operating characteristic (ROC) curve (AUC) and highest accuracy values based on the validation dataset. Finally, six different models were generated, and the models were subsequently evaluated based on the testing dataset for further analysis.

### Statistical Analysis

Follow-up time was defined as the interval from the date of surgery to the date of RM or death from any cause or the last recorded follow-up.

Continuous variables are presented as medians and IQRs, while categorical variables are presented as frequencies and percentages. To analyze differences in patient characteristics among the datasets, one-way analysis of variance was performed for continuous variables (follow-up time), while Pearson χ^2^ test or Fisher exact test was performed for categorical variables.

The mean Dice similarity coefficient (DSC) and 95% Hausdorff distance were used to evaluate the segmentation performance. The AUC with the DeLong test for evaluation of the significant differences and the sensitivity, specificity, accuracy, recall, and Jaccard similarity coefficient were calculated to evaluate the RM status classification performance. The Harrell concordance index (C-index) and area under the time-dependent ROC (time-ROC) curve were used to assess the survival prediction performance. Kaplan-Meier survival analysis with the log-rank test was performed based on the internal and external testing sets to assess the difference in DFS between groups stratified by the predicted risk according to the survival prediction risk score, with a cutoff value of 0.5. To mitigate the potential impact of differences in clinical baseline characteristics between cohorts on model performance, subgroup analyses were performed based on these characteristics to control for this effect. Furthermore, propensity score matching between the training and external testing sets using 1:1 nearest-neighbor matching with a caliper width of 0.2 SD was performed to adjust for the confounding factors.

Statistical analysis was performed using R (version 4.3.0; *http://www.Rproject.org*; packages used are listed in Table S3). *P* values less than .05 were considered statistically significant.

### Code Availability

The modules and packages used in the model construction and statistical analysis are shown in Tables S2 and S3. All source code is publicly available at *https://github.com/run-r/MultiRecNet*.

## Results

### Patient Characteristics and Datasets

After excluding cases with missing or poor-quality images (43 for the primary cohort, 28 for the external cohort), this multicenter study included 445 patients with LARC treated with nCRT at three centers in China (Fig 1). Of these, 373 patients from the primary cohort were used for model construction, hyperparameter optimization, and performance evaluation. Specifically, 261, 37, and 75 patients were randomly assigned to the training (median age, 60 years [IQR, 53–67 years]; 172 male, 89 female), validation (median age, 61 years [IQR, 55–68 years]; 27 male, 10 female), and internal testing (median age, 60 years [IQR, 51–67 years]; 45 male, 30 female) sets, respectively. A total of 72 patients from the external cohort were included as the external testing set (median age, 55 years [IQR, 49–61 years]; 38 male, 34 female).

The characteristics, including baseline clinical and postoperative pathologic data, of each patient were collected (as detailed in the Materials and Methods section), and their distribution difference among training, internal testing, and external testing sets is compared and summarized in Table 1 and Figure S1. Except for location, MR-detected mesorectal fascia, MR-detected extramural venous invasion, pT stage, pN stage, lymphovascular invasion, and postoperative adjuvant chemotherapy, there was no evidence of a difference among the sets. After multiple post hoc comparisons, no evidence of a difference was found between the training and internal testing sets. The external testing set differed from both the training and internal test sets, particularly in location, MR-detected extramural venous invasion, and postoperative adjuvant chemotherapy (*P* < .001).

**Table 1:**
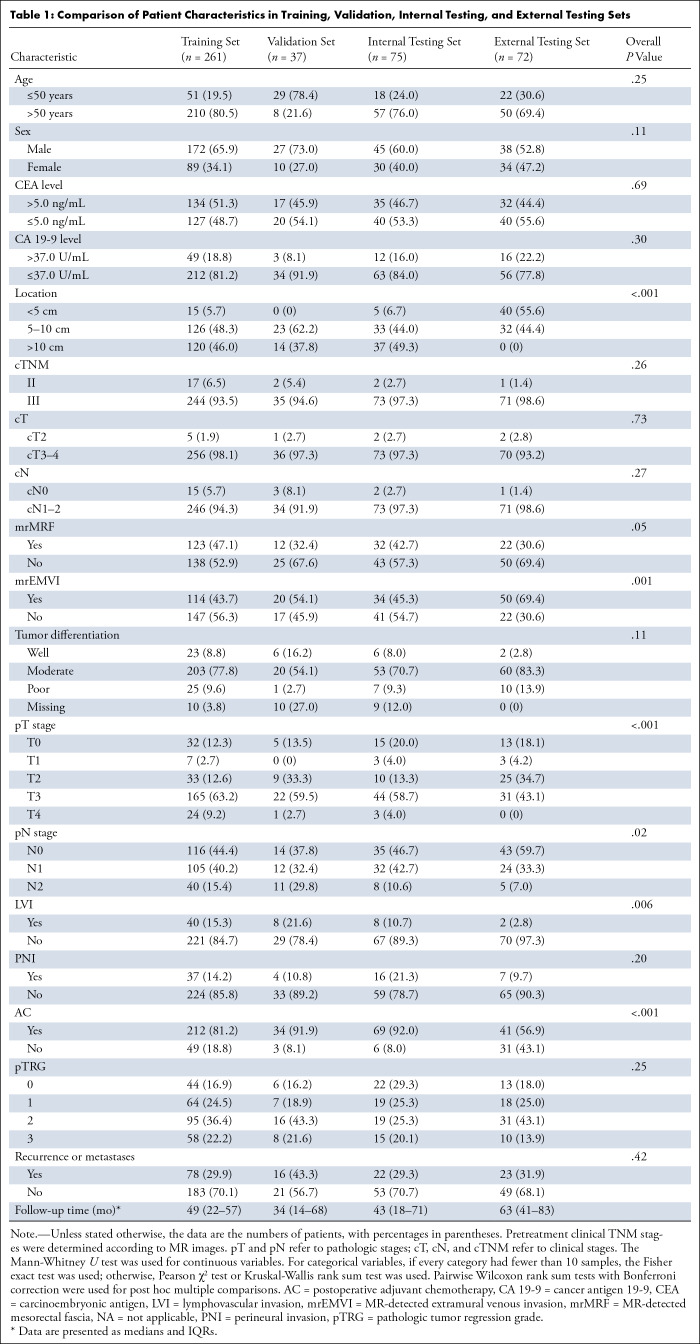
Comparison of Patient Characteristics in Training, Validation, Internal Testing, and External Testing Sets

### Model Segmentation Performance in Internal Testing Set

For models with image data input, the segmentation efficacy of unimodal (the T2 model) and multimodal (the T2+DKI model) image models generated using our network was similar (DSC = 0.69 vs 0.69, *P* > .99), and the segmentation efficacy improved after inputting clinical information into the model (the All model: DSC = 0.72, *P* < .001). The segmentation performance of our network was comparable to models built by single-task networks (Table S4) and emerging multitask networks, including TMSS and DeepMTS, with our model outperforming several state-of-the-art models (Table S5). The detailed results of our models based on the training, internal testing, and external testing sets, as well as the types of input data for each model, are presented in Table 2.

**Table 2:**
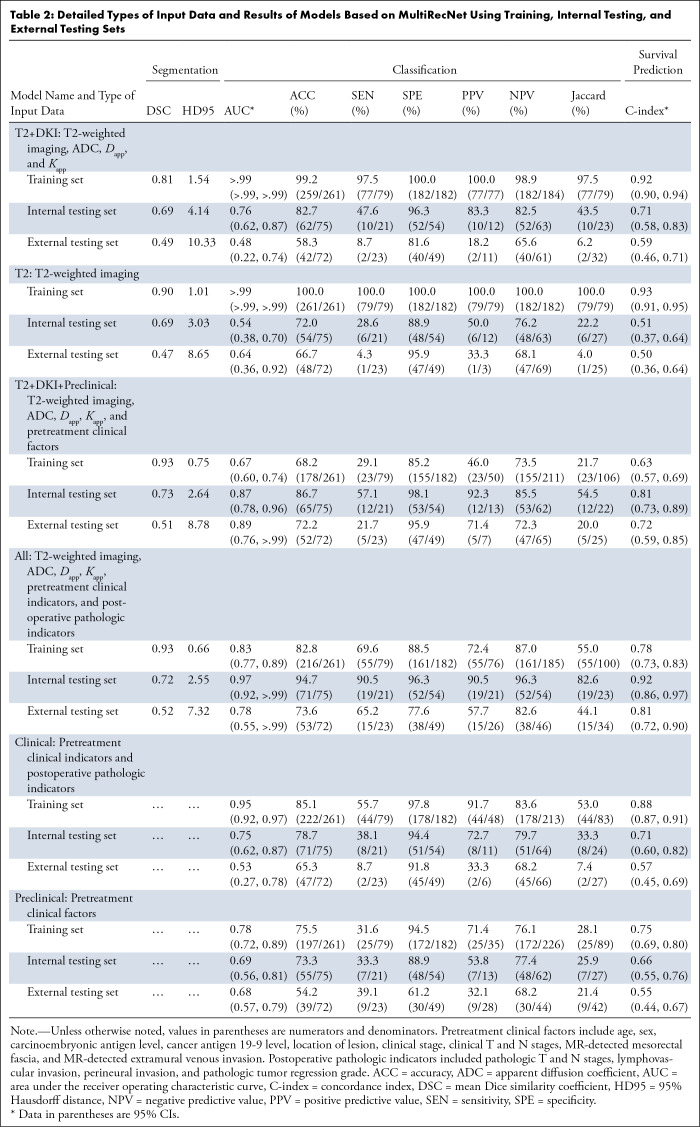
Detailed Types of Input Data and Results of Models Based on MultiRecNet Using Training, Internal Testing, and External Testing Sets

### Model Performance for 3-year RM Classification in Internal Testing Set

**Figure 3:**
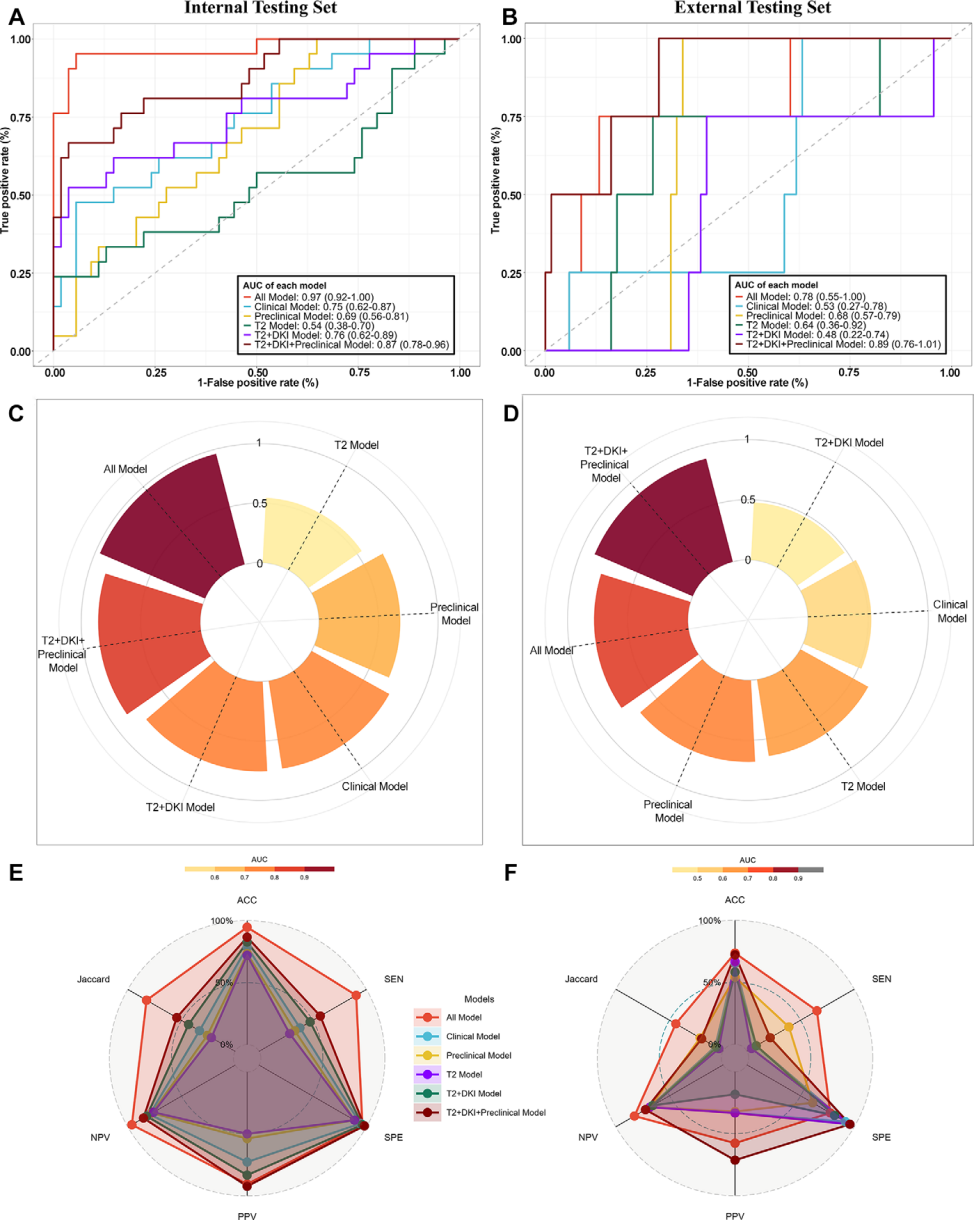
Evaluation of the model’s classification performance receiver operating characteristic (ROC) curves, radar plots of AUC values, and radar plots of other classification evaluation metrics, including the ACC, SEN, SPE, PPV, NPV, and Jaccard value, for RM status classification based on the **(A, C, E)** internal testing and **(B, D, F)** external testing sets. The data in brackets are 95% CIs. ACC = accuracy, AUC = area under the ROC curve, DKI = diffusion kurtosis imaging, NPV = negative predictive value, PPV = positive predictive value, RM = recurrence or metastasis, SEN = sensitivity, SPE = specificity.

For the 3-year RM status classification task, the multimodal image model (T2+DKI model) performed better than the unimodal image model with T2-weighted imaging (T2 model) alone (AUC = 0.76 [95% CI: 0.62, 0.89] vs 0.54 [95% CI: 0.38, 0.70], *P* = .02) and all other conventional convolutional neural networks tested (Table S6), while the model using pretreatment clinical information alone (Preclinical model) underperformed (AUC = 0.69 [95% CI: 0.56, 0.81], *P* > .99 [vs T2+DKI model]). After all the baseline data were merged, the model (T2+DKI+Preclinical model) performance was further improved (AUC = 0.87 [95% CI: 0.78, 0.96], *P* = .40 [vs T2+DKI Model]). When postoperative pathology data were acquired, the model integrating all available data from the baseline and postoperative periods (All model) demonstrated the best performance (AUC = 0.97 [95% CI: 0.92, >.99], *P* = .05 [vs T2+DKI+Preclinical model]), outperforming the traditional model with only clinical information (Clinical model) (AUC = 0.75 [95% CI: 0.62, 0.87], *P* < .001 [vs All model]). The ROC curves are displayed in Figure 3A–3D (Fig S2 for the training set), the decision curve analysis is shown in Figure S3, and other detailed results are shown in Table 2 and Figure 3E and 3F.

### Model-based Stratification of High-Risk Patients for Early RM

**Figure 4:**
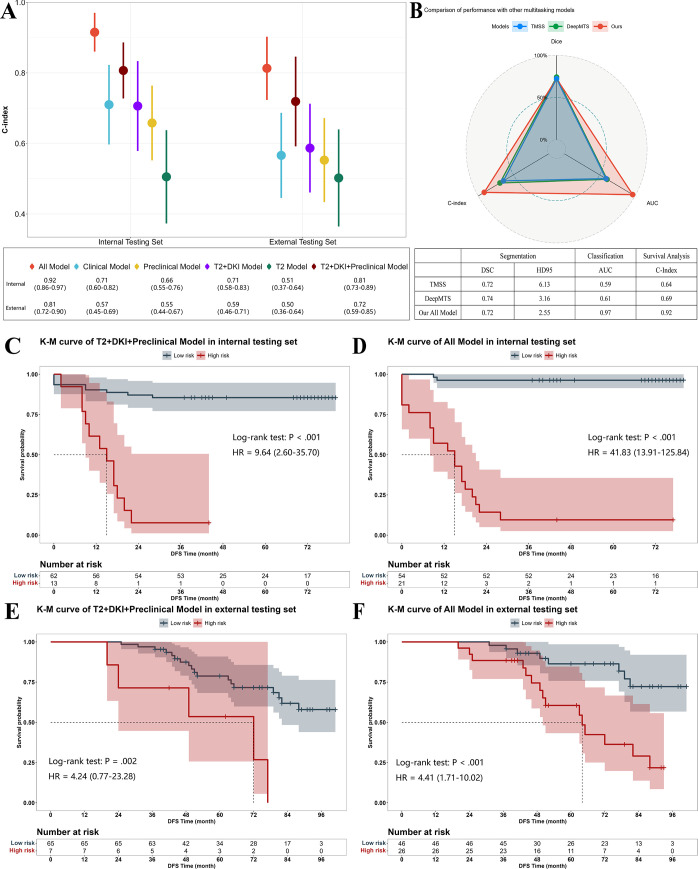
Evaluation of the model’s survival prediction performance. **(A)** Concordance index (C-index) for survival prediction based on models with different data inputs. **(B)** Comparison of the performances of the MultiRecNet All model and other multitask models. Kaplan-Meier (K-M) curves demonstrate differences in the disease-free survival (DFS) distributions between the high-risk (red line) and low-risk (gray line) populations grouped by risk scores calculated from the deep learning models. *P* values were calculated by the log-rank test, and hazard ratios (HRs) were calculated by the likelihood ratio test. **(C)** K-M curves of the T2+DKI+Preclinical model based on the internal testing set. **(D)** K-M curves of the All model based on the internal testing set. **(E)** K-M curves of the T2+DKI+Preclinical model based on the external testing set. **(F)** K-M curves of the All model based on the external testing set. The 95% CIs are presented in parentheses. AUC = area under the receiver operating characteristic curve, DSC = mean Dice similarity coefficient, DKI = diffusion kurtosis imaging, HD95 = 95% Hausdorff distance, T2 = T2-weighted imaging.

For survival prediction, the All model achieved the best performance, with a C-index of 0.92 (95% CI: 0.86, 0.97) for DFS and AUC values for time-ROC of 0.88 (95% CI: 0.78, 0.98), 0.97 (95% CI: 0.92, >.99), and 0.97 (95% CI: 0.92, >.99) for RM status at 1, 2, and 3 years, respectively. Among the multimodal models with only baseline data inputs, the T2+DKI+Preclinical model and the T2+DKI model also demonstrated good performance (C-index: 0.81 [95% CI: 0.73, 0.89] and 0.71 [95% CI: 0.58, 0.83], respectively). The C-index and time-ROC curves of each model are presented in Figures 4A and S4. Compared with other multitask networks, MultiRecNet showed a marked improvement in survival prediction performance (Fig 4B).

Patients were stratified into high- and low-risk groups according to the calculated risk score, and Kaplan-Meier analysis was performed for each model. Except for those in the T2 model, patients in the high-risk group exhibited shorter DFS times than patients in the low-risk group (*P* < .05), and the highest hazard ratios (HRs) for the baseline period and overall were reported for the T2+DKI+Preclinical model (HR = 9.64 [95% CI: 2.60, 35.70], Fig 4C) and the All model (HR = 41.83 [95% CI: 13.91, 125.84], Fig 4D). The Kaplan-Meier curves of the other models are presented in Figure S5.

**Figure 5:**
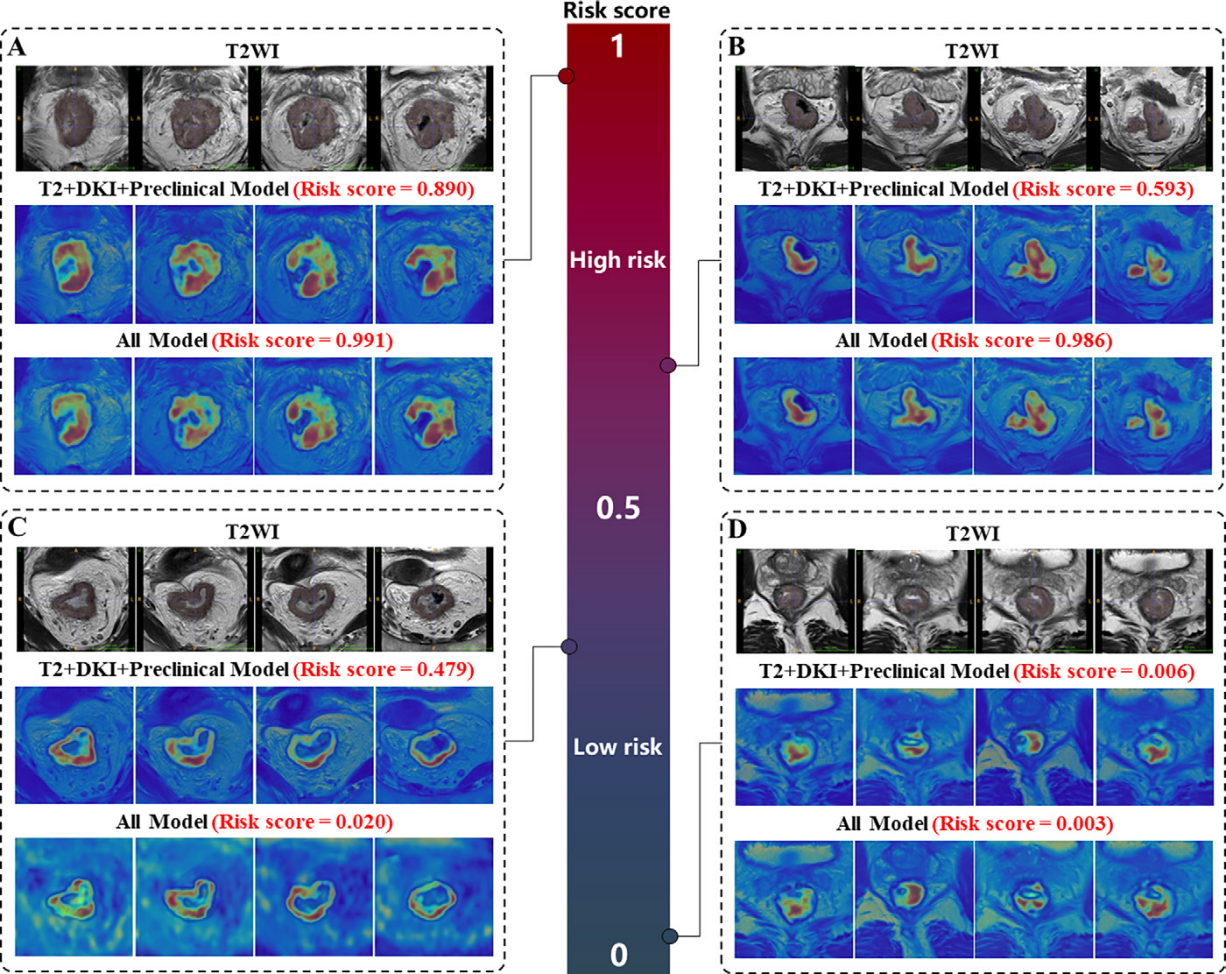
Examples of T2-weighted images with reference standard masks and visualized heatmaps for the T2+DKI+Preclinical model and All model based on MultiRecNet show the regions of the image that were particularly significant for prediction. The closer the risk score calculated by the models is to 1, the greater the risk of early occurrence of RM. **(A)** Images in a 45-year-old male patient with stage cT4aN2 and ypT3N1b disease, pTRG = 3, who underwent AC, with anastomotic recurrence of left seminal vesicle metastasis at 8 months. **(B)** Images in a 38-year-old male patient with stage cT3N2 and ypT3N2a disease, pTRG = 2, who underwent AC, with liver and lung metastases at 20 months. **(C)** Images in a 66-year-old female patient with stage cT4aN2 and ypT3N1a disease, pTRG = 2, who underwent AC, with no RM until the last follow-up at 44 months. **(D)** Images in a 73-year-old male patient with stage cT3aN1b and ypT3N1a disease, pTRG = 2, who underwent AC, with no RM until the last follow-up at 71 months. The attention regions of the MultiRecNet-based models were consistent with the tumor regions. AC = postoperative adjuvant chemotherapy, DKI = diffusion kurtosis imaging, pTRG = pathologic tumor regression grade, RM = recurrence or metastasis, T2WI = T2-weighted imaging.

To visualize the image regions that were particularly for prediction, we employed activation mapping to generate heatmaps (Fig 5A–5D).

### Model Generalizability in External Cohort and Clinical Subgroups

The trends in the classification distribution and survival prediction performance among each model were similar to those determined based on the external testing set, and the best baseline and overall prediction performance were obtained with the T2+DKI+Preclinical model (C-index = 0.72 [95% CI: 0.59, 0.85]) and the All model (C-index = 0.81 [95% CI: 0.72, 0.90]), respectively (Fig 4A). The DFS was greater for the low-risk group than for the high-risk group with the above two models (HR = 4.24 [95% CI: 0.77, 23.28], *P* = .002, Fig 4E; HR = 4.41 [95% CI: 1.71, 10.02], *P* < .001, Fig 4F, respectively).

**Figure 6:**
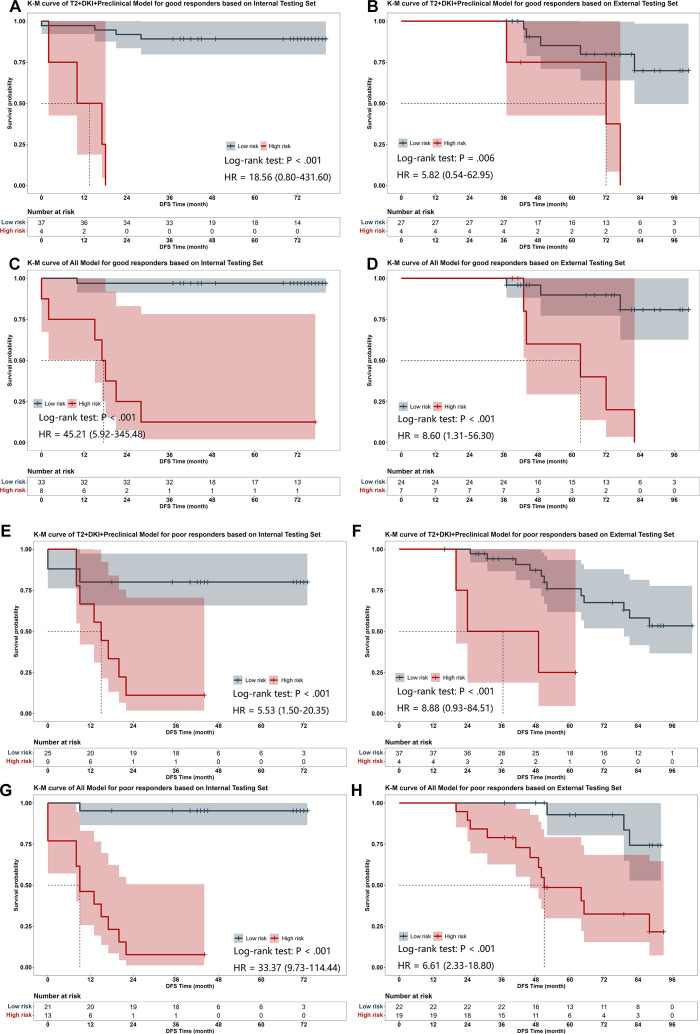
Kaplan-Meier (K-M) curves demonstrate the subgroup analysis of the differences in DFS distributions between the high-risk (red line) and low-risk (gray line) populations, grouped by risk scores calculated from the deep learning models for different treatment responders. A TRG value of 0 or 1 indicates a good responder, while a TRG value of 3 or 4 indicates a poor responder. *P* values were calculated by the log-rank test, and hazard ratios (HRs) were calculated by the likelihood ratio test; the 95% CIs are presented in parentheses. **(A)** K-M curves of the T2+DKI+Preclinical model for good responders based on the internal testing set. **(B)** K-M curves of the T2+DKI+Preclinical model for good responders based on the external testing set. **(C)** K-M curves of the All model for good responders based on the internal testing set. **(D)** K-M curves of the All model for good responders based on the external testing set. **(E)** K-M curves of the T2+DKI+Preclinical model for poor responders based on the internal testing set. **(F)** K-M curves of the T2+DKI+Preclinical model for poor responders based on the external testing set. **(G)** K-M curves of the All model for poor responders based on the internal testing set. **(H)** K-M curves of the All model for poor responders based on the external testing set. DFS = disease-free survival, DKI = diffusion kurtosis imaging, TRG = tumor regression grade, T2 = T2-weighted imaging.

Because the external test set differed from both the training set and the internal test set in terms of location, MR-detected extramural venous invasion, and postoperative adjuvant chemotherapy, subgroup analysis was performed with the external testing set based on these clinical factors. Except for the subgroups without MR-detected extramural venous invasion and without posteroperative adjuvant chemotherapy, the All model could effectively differentiate DFS rates between the high- and low-risk groups in patients of all remaining subgroups (*P* < .01), while the T2+DKI+Preclinical model demonstrated a marked discriminative ability for DFS risk in all subgroups (Fig S6). After adjusting for the tumor location, MR-derived extramural venous invasion, and postoperative adjuvant chemotherapy, the T2+DKI+Preclinical model and All model maintained significant stratification of DFS risk in a propensity score matching–balanced subset (45 matched pairs) of the external testing set (*P* < .001, Fig S7). In addition, the general applicability of the model in populations with different responses to nCRT was validated, and the results showed that the All model and T2+DKI+Preclinical model could effectively differentiate the risk of RM for both good and poor treatment outcomes with both the internal and external testing sets (*P* < .05, Fig 6).

## Discussion

This study developed and validated MultiRecNet, a multitask DL network for predicting RM risk in patients with LARC after nCRT. The network simultaneously performs lesion segmentation, 3-year RM status classification, and survival prediction, automating the clinical prognostic prediction process. MultiRecNet achieved good predictive performance in the initial diagnosis stage (T2+DKI+Preclinical model: AUC = 0.87 [95% CI: 0.78, 0.96], C-index = 0.81 [95% CI: 0.73, 0.89], HR = 9.64 [95% CI: 2.60, 35.70]), with improved performance when including postoperative pathologic indicators (All model: AUC = 0.97 [95% CI: 0.92, >.99], C-index = 0.92 [95% CI: 0.86, 0.97], HR = 41.83 [95% CI: 13.91, 125.84]) in the internal testing set. Its generalizability was validated with an external testing set (C-index = 0.72 [95% CI: 0.59, 0.85] and 0.81 [95% CI: 0.72, 0.90]; HR = 4.24 [95% CI: 0.77, 23.28] and 4.41 [95% CI: 1.71, 10.02] for T2+DKI+Preclinical model and All model, respectively).

Previous studies using traditional radiomic methods and single-task DL methods have achieved good performance in classification and survival prediction tasks, with AUC and C-index values greater than approximately 0.80 (15,18,27–29). However, these methods require labor-intensive manual labeling or use separate networks for segmentation and classification, limiting accuracy and complicating the process. MultiRecNet performs multiple tasks with one network; thus, different tasks can synergize to improve performance and enable a full-process automated prediction pattern. Moreover, the modality-fusion feature allows the model to adapt to various forms of available data in different stages, thereby addressing issues due to lack of some data, which is a common situation in clinical practice.

The ultimate goal of our study was not to accurately segment the tumors, but to predict patient survival. Thus, the necessity of the segmentation task introduced into our study is worth considering. In our pre-experiments (detailed in Appendix S6), we noted that when using the single-task model for each of the three tasks separately, the attention regions identified by the model were similar, which implies potential connections among the three tasks. Thus, the inclusion of the segmentation task could lead to more focused attention regions for both the classification and survival prediction tasks. Another study on multitask modeling for treatment response prediction reached a similar conclusion (30). Although the attention region might not exactly match the area manually labeled by clinicians, the identified area did not deviate from the tumor area. Moreover, our approach may be beneficial for detecting valuable features of the peritumoral area that are not recognized by clinicians, which could improve predictive performance. In fact, several previous studies have demonstrated the relation of imaging features in the peritumor region to tumor biology and clinical outcomes (16,31,32). In our study, MultiRecNet with T2 and DK image inputs performed markedly better than any single-task convolutional neural network with manual labeling in the 3-year RM risk classification task (AUC = 0.76 vs 0.70 [DenseNet, the best-performing convolutional neural network]).

Currently, the choice of subsequent treatment regimens for patients with LARC undergoing nCRT remains controversial and is usually determined according to the response to treatment. However, ignoring the long-term prognosis could lead to an increased risk of over- or undertreatment. In this study, MultiRecNet was used as a prognostic tool. It allows physicians to effectively stratify patients with different risks of RM based on easily accessible clinical and imaging data and to develop more personalized protocols before implementation of the treatment, thus improving the outcome and quality of the patient’s survival. Moreover, a variety of approaches were used in our study to address the censoring of follow-up data, which is a common challenge in survival prediction with DL models. One approach is to transform the survival prediction task into a classification task as was done in several previous studies (27,33,34). This approach was effective for predicting RM status. However, even in populations at higher risk of RM, differences in the duration from treatment to RM were still observed. More aggressive clinical interventions may be necessary for patients predisposed to RM within a short interval after surgery. Therefore, we combined the cross-entropy loss and negative log-likelihood loss for a direct survival analysis task according to the probabilistic nature of the survival data. By using this loss function, we could effectively determine the probability of events occurring over time, accommodating missing data within the analysis. This approach allowed us to interpret model outputs as log-likelihood values, thereby enhancing the interpretability of survival models and enabling more reliable probabilistic predictions. MultiRecNet obtained good results in both tasks, indicating its ability to predict not only the possibility of occurrence but also the risk of early RM.

In addition to improved efficacy, treatment of rectal cancer has increasingly focused on survival time and quality of life. Adopting a nonsurgical “watch and wait” strategy for patients with complete clinical responses could preserve organ function and improve quality of life; however, this strategy requires accurate evaluation (35). Especially for patients who are usually considered to have a better prognosis, such as good responders to nCRT (tumor regression grade = 0 or 1), a conservative treatment strategy is commonly implemented based on the traditional assessment approaches, potentially leading to a poor prognosis for some patients due to insufficient treatment. In our study, the multimodal data inputs to MultiRecNet allowed the prediction task to be performed in different clinical treatment stages while maintaining good predictive performance. Thus, MultiRecNet is effective for both initial treatment protocol selection and determining the risk of early RM after treatment, even if the response is favorable in the current stage (log-rank *P* < .001 in both good and bad responder groups).

Our study had several limitations. First, the retrospective design may have introduced inherent bias and implicit confounding factors. Second, the sample size was small due to the use of the DKI sequence, which is not widely used in clinical practice. However, an improvement in the predictive performance of the model was achieved with the inclusion of the DKI sequence, partly because of its more realistic imaging principle. Third, the small number of patients with certain characteristics may have resulted in nonsignificant differences in survival for some populations in the external testing set. Finally, only one survival metric, that is, the DFS, was analyzed in our study, and overall survival was not investigated due to the small number of events. However, in most previous studies, it was reported that DL-based predictive models performed better for overall survival than for DFS. Thus, our study indicated that the model may be a valid predictor of DFS in patients with LARC.

In conclusion, MultiRecNet demonstrates potential as a preoperative and postoperative risk stratification tool for patients with LARC, supporting the advancement of precision medicine. The network’s ability to fully automatically output risk scores using available multimodal data at different clinical stages warrants further validation in future studies. Future research should focus on prospective validation, incorporation of more conventional imaging sequences, and investigation of additional survival metrics to enhance the model’s clinical applicability and generalizability.

## References

[1] Benson AB , Venook AP , Al-Hawary MM , et al . Rectal Cancer, Version 2.2018, NCCN Clinical Practice Guidelines in Oncology . J Natl Compr Canc Netw 2018 ; 16 ( 7 ): 874 – 901 . 30006429 10.6004/jnccn.2018.0061 PMC10203817

[2] Sineshaw HM , Jemal A , Thomas CR , Mitin T . Changes in treatment patterns for patients with locally advanced rectal cancer in the United States over the past decade: an analysis from the National Cancer Data Base . Cancer 2016 ; 122 ( 13 ): 1996 – 2003 . 27074300 10.1002/cncr.29993

[3] Kolarich A , George TJ , Hughes SJ , et al . Rectal cancer patients younger than 50 years lack a survival benefit from NCCN guideline-directed treatment for stage II and III disease . Cancer 2018 ; 124 ( 17 ): 3510 – 3519 . 29984547 10.1002/cncr.31527 PMC10450543

[4] Denost Q , Fleming CA , Burghgraef T , et al ; Dutch MIRECA Collaborative Group (Pubmed Citable) . An International Multicenter Prospective Study Evaluating the Long-term Oncological Impact of Adjuvant Chemotherapy in ypN+ Rectal Cancer . Ann Surg 2023 ; 277 ( 2 ): 299 – 304 . 36305301 10.1097/SLA.0000000000005742

[5] van Gijn W , Marijnen CAM , Nagtegaal ID , et al ; Dutch Colorectal Cancer Group . Preoperative radiotherapy combined with total mesorectal excision for resectable rectal cancer: 12-year follow-up of the multicentre, randomised controlled TME trial . Lancet Oncol 2011 ; 12 ( 6 ): 575 – 582 . 21596621 10.1016/S1470-2045(11)70097-3

[6] Seo N , Kim H , Cho MS , Lim JS . Response Assessment with MRI after Chemoradiotherapy in Rectal Cancer: current Evidences . Korean J Radiol 2019 ; 20 ( 7 ): 1003 – 1018 . 31270972 10.3348/kjr.2018.0611 PMC6609432

[7] Beets-Tan RGH , Lambregts DMJ , Maas M , et al . Magnetic resonance imaging for clinical management of rectal cancer: updated recommendations from the 2016 European Society of Gastrointestinal and Abdominal Radiology (ESGAR) consensus meeting . Eur Radiol 2018 ; 28 ( 4 ): 1465 – 1475 . 29043428 10.1007/s00330-017-5026-2 PMC5834554

[8] Jayaprakasam VS , Alvarez J , Omer DM , Gollub MJ , Smith JJ , Petkovska I . Watch-and-Wait Approach to Rectal Cancer: the Role of Imaging . Radiology 2023 ; 307 ( 1 ): e221529 . 36880951 10.1148/radiol.221529 PMC10068893

[9] Jensen JH , Helpern JA , Ramani A , Lu H , Kaczynski K . Diffusional kurtosis imaging: the quantification of non-gaussian water diffusion by means of magnetic resonance imaging . Magn Reson Med 2005 ; 53 ( 6 ): 1432 – 1440 . 15906300 10.1002/mrm.20508

[10] Zhu L , Pan Z , Ma Q , et al . Diffusion Kurtosis Imaging Study of Rectal Adenocarcinoma Associated with Histopathologic Prognostic Factors: preliminary Findings . Radiology 2017 ; 284 ( 1 ): 66 – 76 . 27929929 10.1148/radiol.2016160094

[11] Yu J , Xu Q , Song JC , et al . The value of diffusion kurtosis magnetic resonance imaging for assessing treatment response of neoadjuvant chemoradiotherapy in locally advanced rectal cancer . Eur Radiol 2017 ; 27 ( 5 ): 1848 – 1857 . 27631106 10.1007/s00330-016-4529-6

[12] Enkhbaatar NE , Inoue S , Yamamuro H , et al . MR Imaging with Apparent Diffusion Coefficient Histogram Analysis: evaluation of Locally Advanced Rectal Cancer after Chemotherapy and Radiation Therapy . Radiology 2018 ; 288 ( 1 ): 129 – 137 . 29558294 10.1148/radiol.2018171804

[13] Cui Y , Yang X , Du X , Zhuo Z , Xin L , Cheng X . Whole-tumour diffusion kurtosis MR imaging histogram analysis of rectal adenocarcinoma: correlation with clinical pathologic prognostic factors . Eur Radiol 2018 ; 28 ( 4 ): 1485 – 1494 . 29063250 10.1007/s00330-017-5094-3

[14] Zhang XY , Wang L , Zhu HT , et al . Predicting Rectal Cancer Response to Neoadjuvant Chemoradiotherapy Using Deep Learning of Diffusion Kurtosis MRI . Radiology 2020 ; 296 ( 1 ): 56 – 64 . 32315264 10.1148/radiol.2020190936

[15] Cui Y , Wang G , Ren J , et al . Radiomics Features at Multiparametric MRI Predict Disease-Free Survival in Patients With Locally Advanced Rectal Cancer . Acad Radiol 2022 ; 29 ( 8 ): e128 – e138 . 34961658 10.1016/j.acra.2021.11.024

[16] Jiang Y , Jin C , Yu H , et al . Development and Validation of a Deep Learning CT Signature to Predict Survival and Chemotherapy Benefit in Gastric Cancer: a Multicenter, Retrospective Study . Ann Surg 2021 ; 274 ( 6 ): e1153 – e1161 . 31913871 10.1097/SLA.0000000000003778

[17] Hosny A , Parmar C , Coroller TP , et al . Deep learning for lung cancer prognostication: a retrospective multi-cohort radiomics study . PLoS Med 2018 ; 15 ( 11 ): e1002711 . 30500819 10.1371/journal.pmed.1002711 PMC6269088

[18] Jiang X , Zhao H , Saldanha OL , et al . An MRI Deep Learning Model Predicts Outcome in Rectal Cancer . Radiology 2023 ; 307 ( 5 ): e222223 . 37278629 10.1148/radiol.222223

[19] LeCun Y , Bengio Y , Hinton G . Deep learning . Nature 2015 ; 521 ( 7553 ): 436 – 444 . 26017442 10.1038/nature14539

[20] Saeed N , Sobirov I , Majzoub RA , Yaqub M . TMSS: an End-to-End Transformer-Based Multimodal Network for Segmentation and Survival Prediction . In: Wang L , Dou Q , Fletcher PT , Speidel S , Li S , eds. Medical Image Computing and Computer Assisted Intervention (MICCAI) 2022 . Lecture Notes in Computer Science . Vol 13437 . Springer , 2022 : 319 – 329 .

[21] Meng M , Gu B , Bi L , Song S , Feng DD , Kim J . DeepMTS: deep Multi-Task Learning for Survival Prediction in Patients With Advanced Nasopharyngeal Carcinoma Using Pretreatment PET/CT . IEEE J Biomed Health Inform 2022 ; 26 ( 9 ): 4497 – 4507 . 35696469 10.1109/JBHI.2022.3181791

[22] Gu B , Meng M , Xu M , et al . Multi-task deep learning-based radiomic nomogram for prognostic prediction in locoregionally advanced nasopharyngeal carcinoma . Eur J Nucl Med Mol Imaging 2023 ; 50 ( 13 ): 3996 – 4009 . 37596343 10.1007/s00259-023-06399-7 PMC10611876

[23] Collins GS , Reitsma JB , Altman DG , Moons KGM . Transparent Reporting of a multivariable prediction model for Individual Prognosis Or Diagnosis (TRIPOD): the TRIPOD Statement . Br J Surg 2015 ; 102 ( 3 ): 148 – 158 . 25627261 10.1002/bjs.9736

[24] Image Big Data Artificial Intelligence Working Committee of Chinese Society of Radiology Chinese Medical Association; Abdominal Group of Chinese Society of Radiology Chinese Medical Association; Magnetic Resonance Imaging Group of Chinese Society of Radiology Chinese Medical Association . Expert consensus on the colorectal cancer annotation of CT and MRI (2020) [in Chinese] . Chinese Journal of Radiology 2021 ; 55 ( 2 ): 111 – 116 .

[25] Çiçek Ö , Abdulkadir A , Lienkamp SS , Brox T , Ronneberger O . 3D U-Net: learning dense volumetric segmentation from sparse annotation . In: Ourselin S , Joskowicz L , Sabuncu M , Unal G , Wells W , eds. Medical Image Computing and Computer-Assisted Intervention – MICCAI 2016 . Lecture Notes in Computer Science , vol 9901 : 424 – 432 . Springer , Cham .

[26] Scarselli F , Gori M , Tsoi AC , Hagenbuchner M , Monfardini G . The graph neural network model . IEEE Trans Neural Netw 2009 ; 20 ( 1 ): 61 – 80 . 19068426 10.1109/TNN.2008.2005605

[27] Liu Z , Wang Y , Shen F , et al . Radiomics based on readout-segmented echo-planar imaging (RS-EPI) diffusion-weighted imaging (DWI) for prognostic risk stratification of patients with rectal cancer: a two-centre, machine learning study using the framework of predictive, preventive, and personalized medicine . EPMA J 2022 ; 13 ( 4 ): 633 – 647 . 36505889 10.1007/s13167-022-00303-3 PMC9727035

[28] Nie K , Hu P , Zheng J , et al . Incremental Value of Radiomics in 5-Year Overall Survival Prediction for Stage II-III Rectal Cancer . Front Oncol 2022 ; 12 : 779030 . 35847948 10.3389/fonc.2022.779030 PMC9279662

[29] Wang F , Tan BF , Poh SS , et al . Predicting outcomes for locally advanced rectal cancer treated with neoadjuvant chemoradiation with CT-based radiomics . Sci Rep 2022 ; 12 ( 1 ): 6167 . 35418656 10.1038/s41598-022-10175-2 PMC9008122

[30] Jin C , Yu H , Ke J , et al . Predicting treatment response from longitudinal images using multi-task deep learning . Nat Commun 2021 ; 12 ( 1 ): 1851 . 33767170 10.1038/s41467-021-22188-y PMC7994301

[31] Jiang Y , Wang H , Wu J , et al . Noninvasive imaging evaluation of tumor immune microenvironment to predict outcomes in gastric cancer . Ann Oncol 2020 ; 31 ( 6 ): 760 – 768 . 32240794 10.1016/j.annonc.2020.03.295

[32] Braman N , Prasanna P , Whitney J , et al . Association of Peritumoral Radiomics With Tumor Biology and Pathologic Response to Preoperative Targeted Therapy for HER2 (ERBB2)-Positive Breast Cancer . JAMA Netw Open 2019 ; 2 ( 4 ): e192561 . 31002322 10.1001/jamanetworkopen.2019.2561 PMC6481453

[33] Li CH , Cai D , Zhong M-E , et al . Multi-Size Deep Learning Based Preoperative Computed Tomography Signature for Prognosis Prediction of Colorectal Cancer . Front Genet 2022 : 13 : 880093 . 35646105 10.3389/fgene.2022.880093 PMC9133721

[34] Liu X , Zhang D , Liu Z , et al . Deep learning radiomics-based prediction of distant metastasis in patients with locally advanced rectal cancer after neoadjuvant chemoradiotherapy: a multicentre study . EBioMedicine 2021 ; 69 : 103442 . 34157487 10.1016/j.ebiom.2021.103442 PMC8237293

[35] van der Valk MJM , Hilling DE , Bastiaannet E , et al ; IWWD Consortium . Long-term outcomes of clinical complete responders after neoadjuvant treatment for rectal cancer in the International Watch & Wait Database (IWWD): an international multicentre registry study . Lancet 2018 ; 391 ( 10139 ): 2537 – 2545 . 29976470 10.1016/S0140-6736(18)31078-X

